# Computing all hybridization networks for multiple binary phylogenetic input trees

**DOI:** 10.1186/s12859-015-0660-7

**Published:** 2015-07-30

**Authors:** Benjamin Albrecht

**Affiliations:** Institut für Informatik, Ludwig-Maximilians-Universität, 80333München, Amalienstr. 17 Germany

**Keywords:** Hybridization networks, Maximum acyclic agreement forests, Phylogenetics

## Abstract

**Background:**

The computation of phylogenetic trees on the same set of species that are based on different orthologous genes can lead to incongruent trees. One possible explanation for this behavior are interspecific hybridization events recombining genes of different species. An important approach to analyze such events is the computation of hybridization networks.

**Results:**

This work presents the first algorithm computing the hybridization number as well as a set of representative hybridization networks for multiple binary phylogenetic input trees on the same set of taxa. To improve its practical runtime, we show how this algorithm can be parallelized. Moreover, we demonstrate the efficiency of the software Hybroscale, containing an implementation of our algorithm, by comparing it to PIRNv2.0, which is so far the best available software computing the exact hybridization number for multiple binary phylogenetic trees on the same set of taxa. The algorithm is part of the software Hybroscale, which was developed specifically for the investigation of hybridization networks including their computation and visualization. Hybroscale is freely available^1^ and runs on all three major operating systems.

**Conclusion:**

Our simulation study indicates that our approach is on average 100 times faster than PIRNv2.0. Moreover, we show how Hybroscale improves the interpretation of the reported hybridization networks by adding certain features to its graphical representation.

**Electronic supplementary material:**

The online version of this article (doi:10.1186/s12859-015-0660-7) contains supplementary material, which is available to authorized users.

## Background

Recombinational or homoploid hybrid speciation [1] is a mechanism influencing the evolution of species by merging a sizable percentage of the genomes of two different species. It has been discovered especially in plants [2, 3], but also in certain animals [4]. If two individuals each belonging to different species hybridize, a new species, containing genes from both parental individuals, can arise under the following certain circumstances. First of all, the resulting hybrid has to produce viable gametes, which is often a problem due to the two genetically different parental sets of chromosomes preventing a correct meiotic pairing. Second, if these two sets are similar enough and, thus, the hybrid is able to produce any progeny, its early recombinants have to find and successfully colonize its own unexploited niche that is different from either of its parents, which ensures a reduction of the gene flow between its parental genotypes. Due to these circumstances, homoploid hybrid speciation is considered as a rare phenomenon. Note that, after such a new hybrid species has successfully established itself, there can still occur gene flow back from the hybrid species to their parent species, which is denoted as *introgression* [1].

Now, regarding a well-established homoploid hybrid species resulting from such a evolutionary process as described above, we can reconstruct its evolutionary history by taking two different scenarios each corresponding to one of its parental species into account. This is typically done by, first, computing two rooted phylogenetic trees each based on those genes corresponding to one of both parental gene sets and, second, by reconciling these two topologically different trees into one rooted phylogenetic network, whose reticulate nodes (nodes of in-degree ≥2) represent certain putative hybridization events. Because of those major hurdles a hybrid species has to face, hybridization events rarely happen and, thus, from a biological point of view, only those networks containing a minimum number of reticulate nodes are of high interest.

Due to hybridization, the genome of hybrid species, however, can obviously contain more than just two genes having different evolutionary histories. Thus, given a set $\mathcal {T}$ of rooted binary gene trees sharing the same set of taxa, the general problem is to compute a rooted phylogenetic network displaying $\mathcal {T}$ by a minimum hybridization number as defined later by Eq. 2. Unfortunately, this is a well-known *NP-hard* problem, which is however fixed-parameter tractable, even for the simplest case when only just two binary input trees are given [5]. In the general case, however, if the input consists of more than two rooted binary trees, the problem still remains fixed-parameter tractable as recently shown by van Iersel and Linz [6]. More precisely, this means that the problem is exponential in some parameter related to the problem itself, namely the hybridization number, and only polynomial in the size of the input trees. Note that this is an important feature, which facilitates the development of exact algorithms as it is used by our algorithm for some subproblems to maximize efficiency.

In this work, we tackle this *NP-hard* problem by presenting the *first* algorithm that is able to compute the *exact* hybridization number as well as a certain set containing *all* representative networks for, not just only two, but an *arbitrary number* of rooted binary phylogenetic $\mathcal {X}$-trees all sharing the same set of taxa. Note that, until now, the software PIRNv2.0 [7, 8] is the most efficient software that guarantees the computation of the *exact* hybridization number for multiple input trees. In most cases, however, PIRN runs only reasonable efficient if the number of hybridization events is relatively small and, moreover, PIRN does usually output only a small subset of those networks that are computed by our method which plays an important role for the interpretation of the networks as shown later. The algorithm, presented in this work, is based on previous work of Albrecht *et al.* [9] describing an algorithm for just *two* input trees, which itself is based on several works including Baroni *et al.* [10], Bordewich and Semple [11], and Whidden *et al.* [12]. Moreover, this previous approach could only compute a subset of all representative networks and, thus, the motivation for this work was to extend this former algorithm such that now all of those networks for an *arbitrary number* of input trees can be computed.

As we state that our algorithm guarantees the computation of the *exact* hybridization number, we are aware of the fact that this algorithm raises some questions regarding its correctness. However, since in this paper we want to focus on the efficiency of the presented algorithm as well as on the advantages of our software Hybroscale regarding the interpretation of hybridization networks, we decided to discuss those rather complex theoretical issues in a forthcoming paper [13].

Given a hybridization network displaying several input trees, it is often visually challenging for a user to figure out the embedding of those trees. Thus, we have developed the software Hybroscale providing a function for highlighting each input tree by coloring its corresponding edges within a resulting network, which makes it easier for a biologist to analyze hybridization events. Moreover, Hybroscale sorts the set of computed networks by support values indicating how often a certain hybridization event occurs in the set of representative networks.

To demonstrate the efficiency of our implementation, we computed the hybridization number for a specific synthetic dataset and compared the respective runtime with the best currently available software PIRNv2.0 [7, 8]. Note that there are two main differences between our approach and the one corresponding to PIRN. On the one hand, our software provides the better practical runtime for computing hybridization numbers because of parallelization, certain reduction steps, and other algorithmic issues as discussed in the upcoming part of this paper. On the other hand, our approach additionally enables the computation of *all* representative networks allowing the assignment of meaningful support values to each internal node representing a putative hybridization event which helps biologists to figure out hybridization events that might played an important role. Note that the networks reported by PIRN2.0 are either also calculated by our approach or are not considered as being relevant because there exist other networks representing these networks as described in the upcoming part of this paper.

## Methods

In this section, we first introduce the notation and terminology that is used throughout the paper and then present the algorithm ALLHNETWORKS.

### Preliminaries

The upcoming definitions used for describing and discussing our algorithm follow the work of Huson *et al.* [14]. We assume that the user is familiar with general graph-theoretic concepts.

**Phylogenetic trees.** A *rooted phylogenetic* $\mathcal {X}$ *-tree T* is a directed tree, whose edges are directed from the root to the leaves and whose nodes, except the root, have a degree not equal to 2. If *T* is a *binary* tree its root has in-degree 0 and out-degree 2, each inner node an in-degree of 1 and an out-degree of 2, and each leaf an in-degree of 1 and an out-degree of 0. Moreover, each leaf is labeled one-to-one by a taxon of the *taxa set* $\mathcal {X}$, which usually consists of certain species or genes and is also denoted by $\mathcal {L}(T)$. For a node *v* of *T*, the label set $\mathcal {L}(v)$ contains each taxon that is contained in the subtree rooted at *v*. Given a set $\mathcal {F}$ of trees, the label set $\mathcal {L}(\mathcal {F})$ is simply the union of each label set $\mathcal {L}(F_{i})$ of a tree $F_{i}\in \mathcal {F}$.

Now, given a rooted phylogenetic $\mathcal {X}$-tree *T* and a taxa set $\mathcal {X}'\subseteq \mathcal {X}$, we define $T(\mathcal {X}')$ as the minimal connected subgraph of *T* whose leaf set contains each taxon in $\mathcal {X}'$. Additionally, by $T|_{\mathcal {X}'}$ we define the subgraph that is obtained from $T(\mathcal {X}')$ by suppressing all nodes of both in- and out-degree 1. Moreover, given a tree *T*, throughout this paper we use $\overline T$ to denote the tree that is obtained from *T* by suppressing each node of both in- and out-degree 1.

**Fig. 1:**
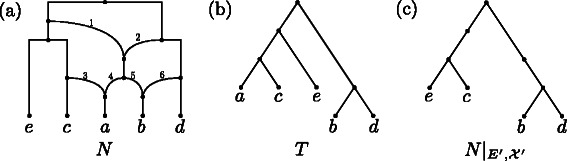
**a** A hybridization network *N* with taxa set $\mathcal {X}=\{a,b,c,d,e\}$ whose reticulation edges are consecutively numbered. **b** A phylogenetic $\mathcal {X}$-tree *T* that is displayed by *N*. Based on *N*, both edge sets {3,6,1} and {3,6,2} refer to *T*. **c** The restricted network $N|_{E',\mathcal {X}'}$ with *E* ^′^={3,6,1} and $\mathcal {X}'=\{b,c,d,e\}$ still containing nodes of both in- and out-degree 1

**Hybridization networks.** A *hybridization network* *N* is a rooted phylogenetic network, which is a rooted acyclic digraph not containing nodes of both in- and out-degree 1 and whose leaves are all labeled one-to-one by a taxon of the taxa set $\mathcal {X}$ (cf. Fig.1(a)). Each node *v* of in-degree greater than 1 is called a *hybridization node* and each edge directed into *v* is called a *reticulation edge* or, in the context of hybridization, a *hybridization edge*. We say a hybridization network *N* on $\mathcal {X}$ *displays* a rooted phylogenetic $\mathcal {X}'$-tree *T* ^′^, with $\mathcal {X}'\subseteq \mathcal {X}$, if we can delete a set of hybridization edges *E* ^′^ followed by suppressing each node of both in- and out-degree 1 such that the resulting rooted phylogenetic $\mathcal {X}$-tree *T* contains *T* ^′^ as restricted subtree on $\mathcal {X}'$. In such a case, we say that *E* ^′^ *refers* to *T* ^′^ (cf. Fig.1(b)). Since a network can display a tree in potentially several ways, *E* ^′^ is not necessarily unique. To quantify the number of reticulation events of a network *N*, the *reticulation number* *r*(*N*) is defined by

(1) $$ r(N)=\sum_{v\in V:\delta^{-}(v)>0}(\delta^{-}(v)-1),  $$

where *δ* ^−^(*v*) denotes the in-degree of node *v*. Moreover, for a set $\mathcal {T}$ of rooted phylogenetic $\mathcal {X}$-trees, we define the *hybridization number* $h(\mathcal {T})$ as

(2) $$ h(\mathcal{T})=\text{min}\{r(N):N~\text{displays each}~T\in\mathcal{T}\}.  $$

Now, given a hybridization network *N* on $\mathcal {X}$ and an edge set *E* ^′^ referring to an embedded rooted phylogenetic $\mathcal {X}'$-tree *T* ^′^ in *N*, the *reduced network* $N|_{E',\mathcal {X}'}$, with $\mathcal {X}'\subseteq \mathcal {X}$, is computed as follows. First, *E* ^′^ is deleted and, second, each node of out-degree 0 that is unlabeled or not labeled by a taxon in $\mathcal {X}'$ is removed repeatedly. The resulting directed graph corresponds to $T'|_{\mathcal {X}'}$ but still contains nodes of both in- and out-degree 1, and, thus, each node in $N|_{E',\mathcal {X}'}$ can be mapped back to exactly one specific node of the unrestricted network *N* (cf. Fig.1(c)). Moreover, the network *N*(*v*) denotes a network rooted at *v* that is computed by, first, removing each node that cannot be reached from *v* and, second, by suppressing each node of both in- and out-degree 1.

**Agreement forests.** Let *T*_1_ and *T*_2_ be two rooted binary phylogenetic $\mathcal {X}$-trees. For technical purpose, we regard the root of both trees *T*_1_ and *T*_2_ as being a node that has been attached to the original roots and to a taxon $\rho \not \in \mathcal {X}$. Now, an *agreement forest* for *T*_1_ and *T*_2_ is a set of components $\mathcal {F}=\{F_{\rho },F_{1},\ldots,F_{k}\}$ on $\mathcal {X} \cup \{\rho \}$ with the following properties.

- Each component *F*_*i*_ with taxa set $\mathcal {X}_{i}$ refers to the restricted subtree $T_{1}|_{\mathcal {X}_{i}}$ and $T_{2}|_{\mathcal {X}_{i}}$, respectively.
- There is exactly one component, denoted as *F*_*ρ*_, containing *ρ*.
- Let $\mathcal {X}_{\rho },\mathcal {X}_{1},\ldots,\mathcal {X}_{k}$ be the taxa sets corresponding to *F*_*ρ*_,*F*_1_,…,*F*_*k*_. Then, all trees in $\{T_{1}(\mathcal {X}_{i})|i\in \{\rho,1,\ldots,k\}\}$ and $\{T_{2}(\mathcal {X}_{i})|i\in \{\rho,1,\ldots,k-1\}\}$ are node disjoint subtrees of *T*_1_ and *T*_2_, respectively.

**Fig. 2:**
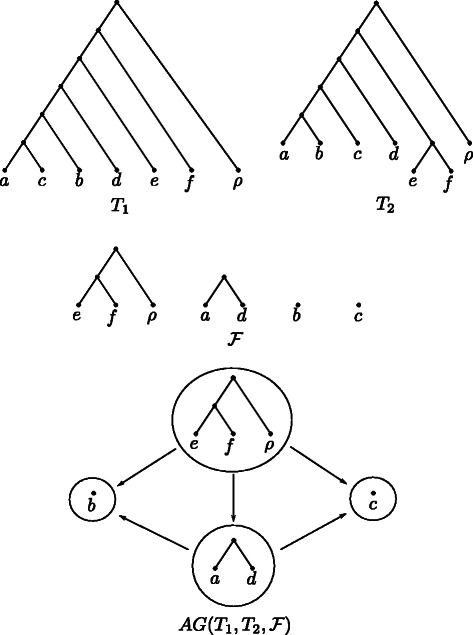
An agreement forest $\mathcal {F}$ of two phylogenetic $\mathcal {X}$-trees *T*_1_ and *T*_2_ together with the corresponding ancestor-descendant graph $AG(T_{1},T_{2},\mathcal {F})$. Note that, as $AG(T_{1},T_{2},\mathcal {F})$ does not contain any directed cycles, $\mathcal {F}$ is acyclic
- the root of $T_{2}(\mathcal {X}_{i})$ is an ancestor of the root of $T_{2}(\mathcal {X}_{j})$,

A *maximum* agreement forest is an agreement forest of minimum size, which implies there does not exist a smaller set of components fulfilling each property listed above. Moreover, we call an agreement forest $\mathcal {F}$ for two rooted binary phylogenetic $\mathcal {X}$-trees *T*_1_ and *T*_2_ *acyclic*, if its underlying *ancestor-descendant graph* $AG(T_{1},T_{2},\mathcal {F})$ does not contain any directed cycles (cf. Fig. 2). More specifically, this graph $AG(T_{1},T_{2},\mathcal {F})$ contains one node corresponding to exactly one component of $\mathcal {F}$. Moreover, two nodes *F*_*i*_ and *F*_*j*_ with *i*≠*j* are connected via a directed edge (*F*_*i*_,*F*_*j*_) if either

- the root of $T_{1}(\mathcal {X}_{i})$ is an ancestor of the root of $T_{1}(\mathcal {X}_{j})$ or

where $\mathcal {X}_{i},\mathcal {X}_{j} \subseteq \mathcal {X}$ denotes the taxa set of *F*_*i*_ and *F*_*j*_, respectively. Again, we call an acyclic agreement forest of minimum size a *maximum acyclic agreement forest*. Note that for a maximum acyclic agreement forest for two rooted binary phylogenetic $\mathcal {X}$-trees *T*_1_ and *T*_2_ containing *k* components there exists a hybridization network whose reticulation number is *k*−1 [15]. This means, in particular, if a maximum acyclic agreement forest for *T*_1_ and *T*_2_ contains only one component, both trees are equal.

If $\mathcal {F}$ is acyclic and, thus, $AG(T_{1},T_{2},\mathcal {F})$ does not contain any directed cycles, one can compute an *acyclic ordering*, as already described in Baroni *et al.* [10], as follows. First, select the node *v*_*ρ*_ of in-degree 0, which corresponds to *F*_*ρ*_, and remove *v*_*ρ*_ by deleting this node together with all its incident edges. Next, again choose a node *v*_1_ with in-degree 0 and remove this node. By continuing this way until finally all nodes have been removed, one receives the ordering *Π*=(*v*_*ρ*_,*v*_1_,…,*v*_*k*_). In the following, we call the ordering of components corresponding to *Π*, denoted by (*F*_*ρ*_,*F*_1_,…,*F*_*k*_), an acyclic ordering of $\mathcal {F}$. As during each of those steps there can occur multiple nodes of in-degree 0, especially if $\mathcal {F}$ contains components only consisting of isolated nodes, such an acyclic ordering is in general not unique.

**Fig. 3:**
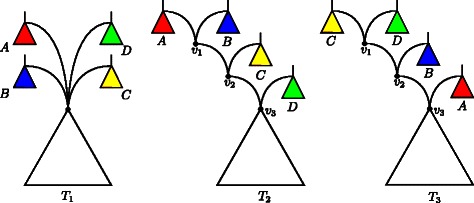
An illustration of a stack of hybridization nodes. The hybridization node with in-degree 4 of the left tree *T*_1_ can be resolved, amongst others, into two different stacks of hybridization nodes (*v*_1_,*v*_2_,*v*_3_) as demonstrated by *T*_2_ and *T*_3_, respectively. Note that by resolving a hybridization node into a stack of hybridization nodes the set of trees that are displayed in the original network remains unchanged

**Representative networks.** As mentioned above, our algorithm ensures the computation of *all representative networks*, which are those hybridization networks with minimum hybridization number (cf. Eq. (2)) fulfilling an additional property that is based on the following observation. Given a hybridization network containing a node *v* with in-degree of at least 3, one can generate further networks by simply dragging some of its hybridization edges upwards resulting in a *stack* of hybridization nodes. More precisely, such a stack is a path (*v*_1_,…,*v*_*n*_) in which each hybridization node *v*_*i*_ is connected through a hybridization edge to *v*_*i*+1_ (cf. Fig. 3). From a biological point of view, such a stack implies that a hybridization event belonging to a hybridization node *v*_*i*_ happened before those corresponding to the in-edges of a hybridization node *v*_*j*_ with *i*<*j*. However, as for each of those networks there exists a network where each stack is fully compressed, we only consider those compressed networks as being relevant.

Consequently, the set of representative networks consists only of those networks with minimum hybridization number not containing any stacks of hybridization nodes leaving the interpretation of the ordering of the hybridization events open. Moreover, just for simplicity, we claim that each of those networks has to be binary not containing any nodes of out-degree greater than 2. By introducing multifurcating nodes, which are nodes having an out-degree of at least 3, the set of representative networks typically shrinks because due to those nodes a network can display several binary networks.

Lastly, given two representative networks *N*_1_ and *N*_2_, we say that *N*_1_ differs from *N*_2_ if either their graph topologies (disregarding edge labels) are not isomorphic or their edge sets indicating the embedding of each input tree differ.

### The algorithm ALLHNETWORKS

In this section, we give a high level description of our algorithm ALLHNETWORKS. More information, involving a more detailed description of the upcoming steps as well as some theoretical issues, will be discussed in a forthcoming paper [13].

The input of the algorithm is a set $\mathcal {T}$ of rooted binary phylogenetic $\mathcal {X}$-trees and its output is either just the hybridization number or all representative networks showing the embedding of those input trees. Similar to the approach described in the work of Albrecht *et al.* [9], ALLHNETWORKS can be separated into three phases. The reduction phase (consisting of a subtree reduction following the work of Bordewich and Semple [16] and a cluster reduction following the work of Baroni *et al.* [10] and Linz [17]), the exhaustive search phase, and the output phase (combining the result of all clusters and undoing each subtree reduction). Whereas the reduction and the output phase can be conducted in polynomial time, the second phase solves an *NP-hard* problem and, thus, its runtime is exponential [5]. However, as recently shown by van Iersel and Linz [6], certain parts of the problem still remain fixed-parameter tractable, which, as already noted in the introduction, is an important feature that is exploited by our algorithm to maximize its efficiency.

At this point, we have to give a remark regarding the correctness of the cluster reduction. The well-known work of Baroni *et al.* [10] contains a proof showing that the *exact* hybridization number of *two* binary phylogenetic $\mathcal {X}$-trees can also be computed by adding up the *exact* hybridization numbers of its minimum common clusters. A more general proof, showing that this concept also holds for *multiple* binary phylogenetic $\mathcal {X}$-trees, can be found in our forthcoming paper [13].

In the upcoming part, we will briefly discuss the exhaustive search phase and its parallelization. A description of the other two phases is omitted but can be looked up in the work of Albrecht *et al.* [9]. The exhaustive search phase runs for an increasing parameter *k* bounding the reticulation number of each computed network. If a hybridization network with reticulation number less than or equal to *k* does not exist, the search is continued with *k*+1 until a hybridization network displaying all input trees can be computed.

**Exhaustive search phase.** Given a set $\mathcal {T}$ consisting of *n* rooted binary phylogenetic $\mathcal {X}$-trees and a parameter $k\in \mathbb {N}$, in a first step we choose an ordering of $\mathcal {T}$, which is for convenience (*T*_1_,*T*_2_,…,*T*_*n*_) in the following. Second, each tree of this ordering is added sequentially to a set $\mathcal {N}$ of networks in all possible ways. At the beginning, $\mathcal {N}$ only consists of the first tree of the ordering, which is *T*_1_ in this case. By adding an upcoming input tree *T*_*i*_(*i*>1), the size of $\mathcal {N}$ grows rapidly, because in general there exist multiple ways of how this can be achieved (cf. Fig. 6). Since we do not delete any edges from a so far computed network *N*, we can disregard those networks whose reticulation number exceeds *k*. Note that, in order to guarantee the computation of all representative networks, this step must be performed for each possible ordering of $\mathcal {T}$.

The input tree *T*_*i*_ is added to a so far computed network *N* by adding hybridization edges connecting certain parts of *N*. Given an edge set *E* ^′^ referring to a phylogenetic $\mathcal {X}$-tree *T* ^′^ that is displayed by *N*, such parts can be derived from the components of a maximum acyclic agreement forest for *T* ^′^ and *T*_*i*_. Again, in order to guarantee the computation of all representative networks, the insertion of *T*_*i*_ has to be performed for all maximum acyclic agreement forests referring to *T*_*i*_ and each phylogenetic $\mathcal {X}$-tree *T* ^′^ that is embedded in *N*, and, additionally, for all edge sets *E* ^′^ referring to *T* ^′^. Note that, given two rooted binary phylogenetic $\mathcal {X}$-trees, the computation of all maximum acyclic agreement forests follows the algorithm ALLMAAFS [18].

A maximum acyclic agreement forest $\mathcal {F}$ of *T*_*i*_ and *T* ^′^ is added to *N* by, first, computing an acyclic ordering (*F*_*ρ*_,*F*_1_,…,*F*_*k*_) of $\mathcal {F}$ which can be done with the help of the directed graph $AG(T',T_{i},\mathcal {F})$ as previously described. Next, each component *F*_*i*_, beginning with *F*_1_, is added to *N* by inserting a new hybridization edge connecting a certain source and target node such that, after all components of $\mathcal {F}$ have been inserted, *N* displays the considered input tree *T*_*i*_. In order to guarantee the computation of the exact hybridization number, all acyclic orderings and all *valid* combinations of source and target nodes, as described below, have to be taken into account. More precisely, in order to avoid directed cycles, we consider a pair (*s*,*t*) of source and target nodes as being *valid* if the source node *s* cannot be reached from *t*. Note that the way of how we add a tree to a network is similar to the algorithm HYBRIDPHYLOGENY [10].

The set of target and source nodes corresponding to a component *F*_*i*_ in $\mathcal {F}$ is defined as follows. Let $\mathcal {F}'=\{F_{\rho },F_{1},\ldots,F_{i-1}\} \subset \mathcal {F}=\{F_{\rho },F_{1},\ldots,F_{k}\}$ be the set of components that has been added so far. Note that, since *N* is initialized with *F*_*ρ*_, at the beginning $\mathcal {L}(\mathcal {F}')$ equals $\mathcal {L}(F_{\rho })$ and the first component that is added is *F*_1_.

*Target Nodes.* The set $\mathcal {V}_{t}$ of target nodes contains all nodes *v* with $\overline N|_{E',\mathcal {L}(\mathcal {F}')\cup \mathcal {L}(F_{i})}(v)$ isomorphic to $T_{i}|_{\mathcal {L}(F_{i})}$. Due to the restriction of the network to $\mathcal {L}(\mathcal {F}')$, this set usually contains more than one node.

*Source Nodes of Type A.* For each edge set *E*_*i*_ referring to the embedded tree $T_{i}|_{\mathcal {L}(\mathcal {F}')}$ in *N*, the set ${\mathcal {V}_{s}^{A}}$ of source nodes of *Type A* contains all nodes *v* with $\overline N|_{E_{i},\mathcal {L}(\mathcal {F}')}(v)$ isomorphic to $T_{i}|_{\mathcal {L}(\mathcal {F}')}(v_{\text {sib}})$, where *v*_sib_ denotes the sibling of the node *v* ^′^ with $\mathcal {L}(v')=\mathcal {L}(F_{i})$ in $T_{i}|_{\mathcal {L}(\mathcal {F}')\cup \mathcal {L}(F_{i})}$. Note that, due to the restriction of the network to $\mathcal {L}(\mathcal {F}')$, this set usually consists of more than one node.

*Source Nodes of Type B.* The set ${\mathcal {V}_{s}^{B}}$ of source nodes of *Type B* is computed such that it contains each node *v* of a subtree, whose root is a sibling of a node in ${\mathcal {V}_{s}^{A}}$ and which does not contain any taxa of $\mathcal {L}(\mathcal {F}')$. Moreover, its leaf set $\mathcal {L}(v)$ has to consist only of several subsets representing the total taxa set $\mathcal {L}(F)$ of a component *F* in $\mathcal {F}$, which means that *v* must not be part of a subtree corresponding to a component that is added afterward.

**Fig. 4:**
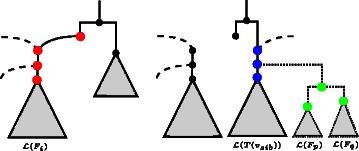
An illustration of the definitions of target (left) and source nodes (right) for a component *F*_*i*_ (*p*,*q*>*i*) in which red nodes correspond to target nodes, blue nodes to source nodes of *Type A*, and green nodes to source nodes of *Type B*. Moreover, dashed edges and dotted edges are those edges that are disregarded when considering the restricted network in terms of the chosen embedded tree and the taxa set of the so far added components, respectively

For a better understanding the definitions of source and target nodes are illustrated in Fig. 4.

**Fig. 5:**
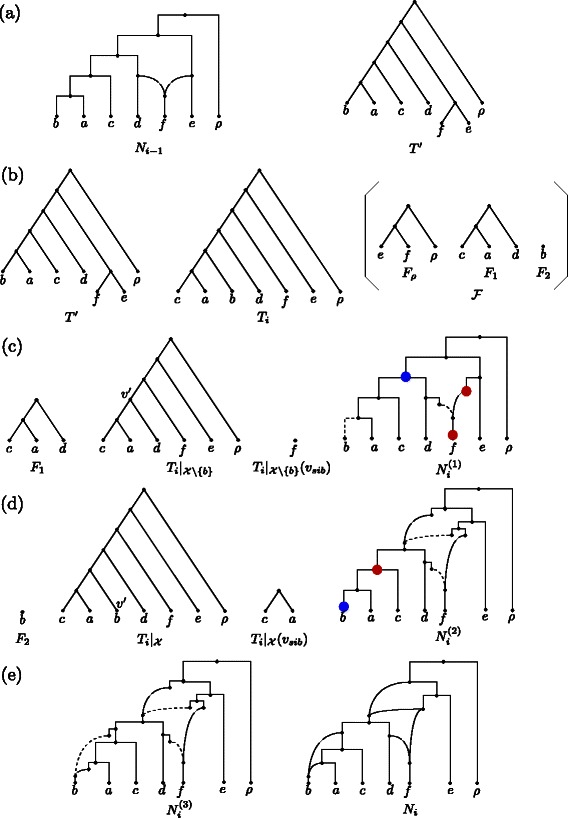
An illustration of how an input tree *T*_*i*_ is inserted into a network *N*_*i*−1_ with the help of an embedded tree *T* ^′^. **a** The network *N*_*i*−1_ together with an embedded tree *T* ^′^. **b** The input tree *T*_*i*_, which will be embedded into *N*_*i*−1_ by inserting the maximum acyclic agreement $\mathcal {F}$ forest of *T*_*i*_ and *T* ^′^ consisting of three components *F*_*ρ*_, *F*_1_, and *F*_2_. **c**, **d** All important elements that have to be considered during the insertion of both components *F*_1_ and *F*_2_, respectively. Blue dots correspond to source nodes and red nodes to target nodes. Note that, regarding $N_{i}^{(1)}$, there is only one valid pair of source and target nodes. Dashed edges are those edges that are disregarded when considering the restricted network in terms of *T* ^′^ and the taxa set of the so far added components of $\mathcal {F}$. **e** The resulting network *N*_*i*_, which is obtained from $N_{i}^{(3)}$ by suppressing each node of both in- and out-degree 1

Now, given a valid pair (*s*,*t*) of source and target nodes, a new hybridization edge is inserted as follows. The in-edge of the source node *s* is split in a way that there is a new node *s* ^′^ that is connected to *s* and to the parent of *s*. If the parent of *t* is of in-degree one the in-edge of *t* is split in the same way. Otherwise, its parent node acts as *t* ^′^ which allows the computation of networks containing nodes of in-degree greater than 2. Note that this is an optional step that is necessary to ensure that each computed hybridization network does not contain any stacks of hybridization nodes such that it applies to the definition of a representative network. Finally, the two nodes *s* ^′^ and *t* ^′^ are connected through a path *P* consisting of two edges. This is done because, on the one hand, we only allow nodes of in-degree one as source nodes, but, on the other hand, in order to compute all representative networks, we have to enable that a target node can additionally be attached to hybridization edges. Due to this fact, however, before reporting a network embedding all input trees, one still has to suppress all nodes of both in- and out-degree 1. By referring to the terminology used above, in Fig. 5 we give a short example of how a certain input tree is added to a network.

For clarity, the algorithm ALLHNETWORKS has been described so far with respect to one single embedding of each input tree. Given *n* input trees, to generate all representative networks, one still has to compute for each network all possible combinations of edge sets each referring to the embedding of one input tree.

Lastly, we give some high level ideas why the algorithm ALLHNETWORKS is correct, i.e., calculates all representative networks for a set of rooted binary phylogenetic $\mathcal {X}$-trees. We refer readers who are interested in a detailed proof to our forthcoming paper [13]. Let *N* ^′^ be a representative network displaying a subset $\mathcal {T}'$ of all input trees $\mathcal {T}$ and let *N* be a hybridization network (not containing any stacks of hybridization nodes) that is based on *N* ^′^ and displays a further input tree $T_{i}\not \in \mathcal {T}$. Then, one can obtain *N* from *N* ^′^ by inserting a set *E* ^′^ of reticulation edges whose source and target nodes can be derived from an acyclic agreement forest $\mathcal {F}$ for *T*_*i*_ and a certain tree displayed by *N* ^′^. This is due to the fact that *N* ^′^ must contain such an agreement forest $\mathcal {F}$ so that each of its components, except *F*_*ρ*_, is rooted at a target node of an edge in *E* ^′^ whose incident nodes are contained in $\mathcal {V}_{t}$ and ${\mathcal {V}_{s}^{A}}{\cup \mathcal {V}_{s}^{B}}$, respectively. Moreover, one can show that by constructing networks for all possible orderings of the input trees, it suffices to take only maximum acyclic agreement forests into account.

### Parallelization

**Fig. 6:**
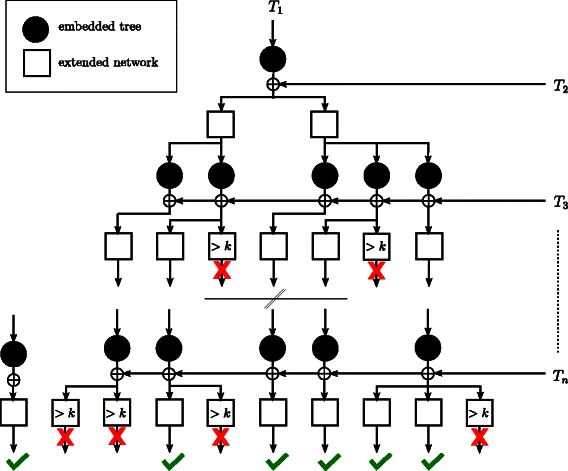
An illustration of how the insertion of the input trees is conducted by the algorithm ALLHNETWORKS in respect of the parameter *k* bounding the maximal reticulation number of resulting networks. Beginning with the first input tree *T*_1_, repeatedly, first, an embedded tree *T* ^′^ of a so far computed network *N* is extracted, and, second, the current input tree *T*_*i*_ is inserted into *N* by sequentially adding the components of a maximum acyclic agreement forest for *T* ^′^ and *T*_*i*_. As soon as the reticulation number of a so far computed network exceeds *k* one can be sure that this network cannot lead to a network with reticulation number smaller or equal to *k* and, thus, the corresponding computational path can be aborted

In order to improve the practical runtime of the algorithm ALLHNETWORKS, our implementation is able to run the exhaustive search looking for hybridization networks with hybridization number *k* in parallel. As mentioned above, the insertion of an input tree *T*_*i*_ to a so far computed network results in several new networks, which are then processed by inserting the next input tree *T*_*i*+1_ of the chosen ordering (cf. Fig. 6). Since the processing of networks runs independently from each other, these steps can be parallelized in a simple manner. Based on the number of hybridization edges of a so far computed network, each of those steps is more or less likely to result in a representative network. Thus, we set up a priority queue to process the most promising networks first, which, on the one hand, depends on the number of so far inserted input trees and, on the other hand, on its reticulation number.

Such a priority queue, however, does only speed up the computation of the hybridization number, since in this case all computational paths can be aborted immediately as far as the first minimum hybridization network could be computed successfully. For the computation of all representative networks, however, each computational path has to be processed anyway until either it can be early aborted (which is the case if the reticulation number of the corresponding network exceeds *k*) or it leads to a representative network.

Moreover, as the algorithm computes networks for all different orderings of input trees and all different acyclic orderings of maximum acyclic agreement forests, a representative network can be computed multiple times. As a consequence, to ensure that the output only consists of unique networks, one has to filter the set of networks obtained from the exhaustive search step. For this purpose, we first group this set after the sum of *support values* computed for each network (as defined later) and then check each of those subgroups for isomorphic networks in parallel. Due to the typically large number of computed networks (cf. Tables 1, 2), the restriction of the filtering step to small subgroups usually provokes a large speedup. Note that, as already mentioned above, we consider two networks as being different if either their graph topologies (disregarding edge labels) are not isomorphic or their sets of edges that are necessary for displaying each input tree differ.

### Additional features

**Fig. 7:**
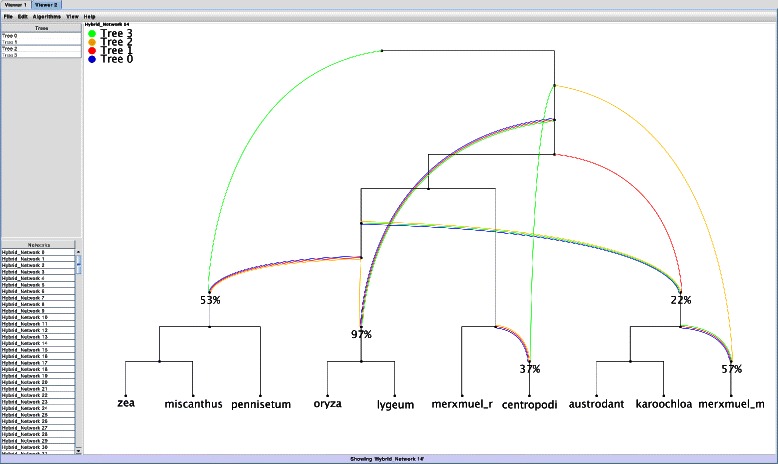
Our software Hybroscale showing a hybridization network displaying the embedding of four input trees by the colors blue, red, green, and orange

Given just the *extended newick format* [19] of a hybridization network, its topology is in general hard to interpret. Although there exist software packages, which are able to display rooted phylogenetic networks, e.g., the software Dendroscope [14], most of them are not able to visualize the embedding of all input trees, which is a preferable feature for studying hybridization events. In order to close this gap, we have developed the software Hybroscale, which is specifically designed for studying hybridization networks. Besides the computation of a graphical layout of rooted trees and rooted networks, which is optimized by minimizing the number of crossings between all hybridization edges, Hybroscale can additionally highlight each hybridization edge that is necessary for displaying all embedded input trees by assigning a specific color to each tree (cf. Fig 7). Thus, Hybroscale is a software that, on the one hand, enables an easy handling of our algorithm and, on the other hand, ensures the readability of the computed networks.

Furthermore, Hybroscale assigns each hybridization node a *support value* indicating the fraction of networks containing this node and, additionally, sorts the reported networks by the sum of those values in decreasing order. More specifically, the computation of support values is done as follows. Given a network *N*, each edge set *E*_*i*_ referring to one of the input trees *T*_*i*_, and a certain hybridization node *v*, we, first, compute the following ordering of taxa sets $\Pi (v)=(\mathcal {L}(N(v)|_{E_{1},\mathcal {X}}), \ldots,\mathcal {L}(N(v)|_{E_{n},\mathcal {X}}))$. More precisely, each element $\mathcal {L}(N(v)|_{E_{i},\mathcal {X}})$ consists of those taxa adhering to each leaf that can be reached from *v* by directed paths only crossing those hybridization edges in *E*_*i*_ indicating the embedding of *T*_*i*_. For example, regarding Fig. 7, the set referring to the hybridization edges indicating the embedding of *Tree 2* and the node labeled by 22 *%* is {*a* *u* *s* *t* *r* *o* *d* *a* *n* *t*,*k* *a* *r* *o* *o* *c* *h* *l* *o* *a*}. Second, we determine the fraction of networks containing *Π*(*v*). This step ensures that the user can instantly look at those networks containing the most promising hybridization events, which is an important feature, because usually a large number of networks is reported (cf. Tables 1, 2).

**Table 1:** Output produced by Hybroscale applied to *two* phylogenetic trees belonging to a well known grass (*Poaceae*) dataset

Genes	Taxa	HNumber	#MAAFs	#HNetworks
ndhf phyB	40	8	459	2079
ndhf rbcl	36	8	72	1488
ndhf rpoc	34	9	144	264
ndhf waxy	19	6	46	599
phyB its	30	8	21	195
phyB rbcl	21	4	4	6
phyB rpoc	21	4	5	9
phyB waxy	14	3	6	10
rbcl rpoc	26	7	18	111
rbcl waxy	12	4	10	84
rpoc its	31	12	12	3480
rpoc waxy	10	2	1	1
waxy its	15	5	6	15

**Table 2:** Output produced by Hybroscale applied to phylogenetic trees belonging to a grass (*Poaceae*) dataset. Each runtime given in this table is stated in seconds. A missing result for a certain tree set means that our software Hybroscale could not compute the *exact* hybridization number (resp. set of representative networks) within 20 minutes

		Computing HNumbers		Computing HNetworks	
Genes	#Taxa	HNumber	Runtime	#HNetworks	Runtime
ndhf its	46	17	3.262	-	-
ndhf phyB	40	8	0.199	2079	33.035
ndhf rbcl	36	8	0.175	1488	32.1
ndhf rpoc	34	9	0.197	264	5.353
ndhf waxy	19	6	0.179	599	5.693
phyB its	30	8	0.238	195	8.304
phyB rbcl	21	4	0.083	6	1.65
phyB rpoc	21	4	0.091	9	1.678
phyB waxy	14	3	0.071	10	1.615
rbcl its	29	12	4.41	-	-
rbcl rpoc	26	7	0.147	111	3.836
rbcl waxy	12	4	0.126	84	4.338
rpoc its	31	12	4.5	3480	217.575
rpoc waxy	10	2	0.07	1	1.582
waxy its	15	5	0.118	15	2.712
ndhf phyB its	30	13	243.411	-	-
ndhf phyB rbcl	21	9	7.226	-	-
ndhf phyB rpoc	21	8	6.189	36948	206.114
ndhf phyB waxy	14	4	1.599	54	2.87
ndhf rbcl its	28	-	-	-	-
ndhf rbcl rpoc	26	11	7.223	46946	511.296
ndhf rbcl waxy	12	5	4.198	114	5.577
ndhf rpoc its	31	-	-	-	-
ndhf rpoc waxy	10	3	2.583	14	2.632
ndhf waxy its	15	8	4.213	6490	26.697
phyB rbcl its	17	8	9.437	8661	233.768
phyB rbcl rpoc	15	6	4.652	40	4.867
phyB rbcl waxy	7	2	2.568	11	2.592
phyB rpoc its	19	7	3.774	57	4.633
phyB rpoc waxy	5	0	0.045	1	0.075
phyB waxy its	10	4	3.122	204	3.844
rbcl rpoc its	24	-	-	-	-
rbcl rpoc waxy	9	3	1.585	5	1.62
rbcl waxy its	11	6	6.224	63	7.49
rpoc waxy its	10	4	2.626	4	2.635
ndhf phyB rbcl its	17	-	-	-	-
ndhf phyB rbcl rpoc	15	9	224.934	1517	403.728
ndhf phyB rbcl waxy	7	2	2.581	1	2.594
ndhf phyB rpoc its	19	9	984.937	-	-
ndhf phyB rpoc waxy	5	0	0.056	1	0.071
ndhf phyB waxy its	10	5	4.159	8016	26.434
ndhf rbcl rpoc its	24	-	-	-	-
ndhf rbcl rpoc waxy	9	4	3.165	396	14.864
ndhf rbcl waxy its	11	6	54.399	2	159.99
ndhf rpoc waxy its	10	5	4.213	324	16.663
phyB rbcl rpoc its	14	-	-	-	-
phyB rbcl rpoc waxy	4	0	0.057	1	0.06
phyB rbcl waxy its	6	2	2.574	3	2.589
phyB rpoc waxy its	5	0	0.064	1	0.065
rbcl rpoc waxy its	9	5	7.205	333	38.471
ndhf phyB rbcl rpoc its	14	-	-	-	-
ndhf phyB rbcl rpoc waxy	4	0	0.066	1	0.084
ndhf phyB rbcl waxy its	6	3	4.232	135	22.506
ndhf phyB rpoc waxy its	5	0	0.059	1	0.083
ndhf rbcl rpoc waxy its	9	5	35.899	235	587.54
phyB rbcl rpoc waxy its	4	0	0.066	1	0.076
ndhf phyB rbcl rpoc waxy its	4	0	0.062	1	0.083

## Results and discussion

In this section, we first report a simulation study indicating that our approach is much faster than other existing methods and then illustrate how Hybroscale can be used for studying hybridization networks by applying the software to a well known grass (*Poaceae*) dataset.

### Simulation study

To show the efficiency of our implementation, we have integrated our algorithm into the Java software Hybroscale and conducted a simulation study comparing its runtime to PIRNv2.0 [7, 8], which is so far the best available software for computing exact hybridization numbers for multiple rooted binary phylogenetic $\mathcal {X}$-trees.

Our synthetic dataset is freely available^2^ and consists of several tree sets each containing multiple rooted phylogenetic $\mathcal {X}$-trees. Each $\mathcal {X}$-tree is generated by ranging over all different combinations of four parameters, namely the number of input trees *n*, the number of leaves *ℓ*, an upper bound for the hybridization number *k*, and the *cluster degree* *c* as defined below. Each of the *n* input trees is obtained from a bicombining network *N*, which means that *N* only contains hybridization nodes of in-degree 2. This network *N* is computed in respect to these four different parameters as follows. In a first step, a random binary tree *T* with *ℓ* leaves is computed which is done in the following way. First, at the beginning, two nodes *u* and *v* of a specific set *V*, which is initialized by *ℓ* nodes of both in- and out-degree 0, are randomly selected. Those two selected nodes *u* and *v* are then connected to a new node *w* and, finally, *V* is updated by replacing *u* and *v* by its parent node *w*. This process is repeated until *V* consists only of one node corresponding to the root of *T*. In a second step, *k* hybridization edges are created in *T* with respect to parameter *c* such that the resulting network *N* contains exactly *k* hybridization nodes of in-degree 2.

**Fig. 8:**
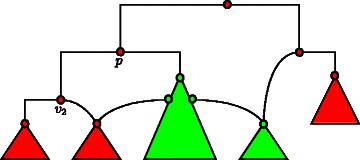
An illustration of the *cluster degree* parameter *c*=1. When inserting an in-going edge *e* to node *v*_2_ that is respecting *c*, each node that is marked green or is part of a green marked subnetwork forms a potential source node

In this context, the *cluster degree* is an *ad hoc* concept influencing the computational complexity of a tree set similar to the concept of the *tangling degree* introduced in the work of Scornavacca *et al.* [18]. When adding a hybridization edge *e* with target node *v*_2_ and source node *v*_1_, we say that *e* respects cluster degree *c*, if *v*_1_ cannot be reached from *v*_2_ and there is a path of length less than or equal to *c* leading from *v*_2_ to a certain node *p* such that *v*_1_ can be reached from *p*. Consequently, networks providing a small cluster degree in general contain more minimum common clusters than networks of large cluster degrees and, thus, typically can be processed quite fast when applying a cluster reduction beforehand. For a better understanding, in Fig. 8 an example of this concept is depicted.

**Fig. 9:**
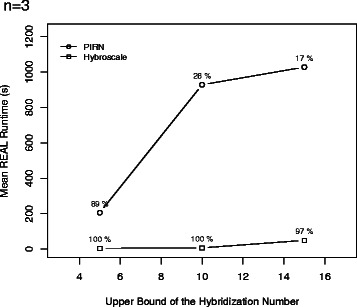
The figure shows the mean average runtime corresponding to Hybroscale and PIRNv2.0 grouped by parameter *k* denoting the hybridization number of the network that was used to obtain the tree set $\mathcal {T}$ from. Thus, this parameter *k* acts as an upper bound of the hybridization number of $\mathcal {T}$. Each percentage indicates the proportion of tree sets that could be computed within the time limit of 20 minutes

**Fig. 10:**
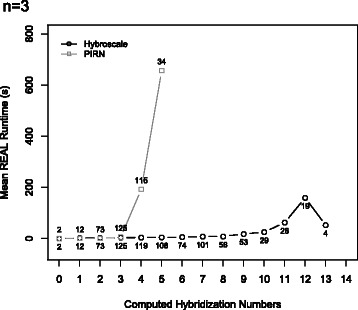
The figure shows the mean average runtime of all tree sets grouped by the computed hybridization numbers. The numbers inside the plot indicate how many tree sets could be computed for the corresponding hybridization number within the time limit of 20 minutes. Note that for the hybridization numbers 0 to 3 all corresponding tree sets could be computed by Hybroscale and PIRNv2.0 within comparable runtimes

**Fig. 11:**
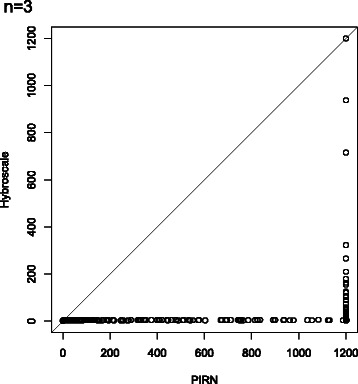
A scatterplot of the runtimes generated by PIRNv2.0 (*x*-axis) against the runtimes generated by Hybroscale (*y*-axis) of all 810 data sets consisting of *three* input trees. Note that PIRNv2.0 is not able to compute the result for 449 tree sets corresponding to each dot in the figure whose *x*-value is 1200. From those tree sets just 6 according to the dots whose *y*-value is also 1200 could not be computed by Hybroscale

**Fig. 12:**
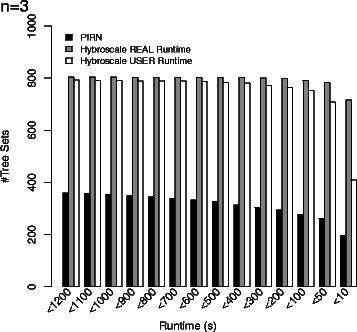
The figure shows the number of tree sets that could be computed within the runtime given on the *x*-axis by considering the real-runtime of PIRN and both real- and user-runtime of Hybroscale. Only the rightmost bar group reveals that the massive parallelization with 16 cores can significantly improve the runtime of Hybroscale in this case. Note that this is, on the one hand, due to the low time limit of just 20 minutes and, on the other hand, due to the low computational complexity of the considered tree sets

**Fig. 13:**
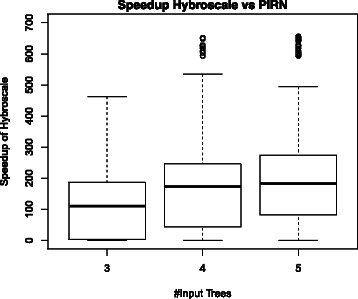
Distribution of the speedups of Hybroscale versus PIRN computed for each tree set of our synthetic dataset. For three input trees Hybroscale is on mean average about 110 times faster than PIRN, for four input trees on mean average about 170 times, and for five input trees on mean average about 190 times

To compare the efficiency, both programs have been run on a grid computer providing 16 cores and 40 GB RAM for our synthetic dataset containing tree sets with parameters *n*∈{3,4,5}, *ℓ*∈{10,25,50}, *k*∈{5,10,15}, and *c*∈{1,3,5}. More precisely, we have generated for all 81 combinations of the four parameters 30 tree sets as described above resulting in 2430 tree sets in total. The results for three input trees (*n*=3) are presented in Figs. 9, 10 and 11, whereas the results for four and five input trees (*n*=4,5) can be found in the Additional file 1. Due to time limitations, if the hybridization number of a certain tree set could not be computed within 20 minutes, the computation of this tree set was aborted. In Figs. 9, 11, 12, and 13 those unfinished tree sets were taken into account with a runtime of 20 minutes whereas in Fig. 10 these tree sets were omitted.

Each of the simulation results given in Figs. 9, 10, 11, 12, and 13, which are now discussed in more detail, clearly demonstrates that our implementation is much faster than PIRN.

Figure 9 shows that, by increasing the upper bound of the hybridization number *k*, the mean average runtime of the datasets computed by PIRN increases up to 1000 seconds whereas the mean average runtime corresponding to Hybroscale is always below 100 seconds. Note that, as the runtime of each unfinished dataset was set to 1200 seconds, if we would set the time limit to a higher value, the maximal mean average runtime produced by PIRN is expected to be even higher — otherwise, to produce a reasonable comparison between both programs, we would have to leave out each dataset, which could not be computed by one of both programs, which means that we would end up with only those non representative datasets that are quite easy to compute. Figure 10 shows that Hybroscale, in comparison to PIRN, can compute more datasets within the time limit and datasets having a significant larger hybridization number. Whereas PIRN is just able to compute hybridization numbers up to 5, Hybroscale is able to compute hybridization numbers up to 13.

Thus, it is obvious that our implementation outperforms PIRN which becomes even clearer by looking at Fig. 11 showing a scatterplot of the runtimes produced by both programs. The figure shows that for each runtime of a specific data set produced by PIRN the corresponding runtime of Hybroscale is smaller or equal. Moreover, looking at the bottom right of the figure, there exist a lot of data sets that could be computed by Hybroscale quite fast in less than 200 seconds, whereas PIRN is not able to come up with a result in less than 1200 seconds.

By comparing real- with user-runtimes, Fig. 12 demonstrates that the better performance of Hybroscale is not only due to the applied massive parallelization. As the user-runtime indicates the total CPU time, which means that the time spent on all available cores is simply added up, this time indication corresponds to the runtime produced by a program that is executed on a system only providing a single core with no parallel execution taking place. Figure 12 shows the number of tree sets that could be computed within the runtime given at the *x*-axis. For example, the leftmost bar-group shows that PIRN could only finish 360 of 810 tree sets consisting of three trees within 1200 seconds whereas Hybroscale could finish 804 by taking parallelization into account and 793 by not taking parallelization into account. Note that the difference between both bars corresponding to the real- and user-runtime of Hybroscale would be even larger if, on the one hand, the dataset would contain tree sets of higher computational complexity and, on the other hand, the time limit would be set to a higher value. A possible explanation for the speedup without taking advantage of parallelization is, on the one hand, the proven method allMAAFs [18] that is used for solving the *NP-hard* problem of computing all maximum acyclic agreement forests. The efficiency of this method has been indicated recently in the work of Albrecht *et al.* [9]. On the other hand, in contrast to our approach, we assume that PIRN does only apply a subtree reduction and not additionally a cluster reduction to the set of initial input trees.

Finally, we have computed the speedup of Hybroscale versus PIRN by comparing its runtimes produced for each tree set within our synthetic dataset. More precisely, for each tree set *d* we have computed the speedup *s*(*d*)=*R*_*P*_(*d*)/*R*_*H*_(*d*), where *R*_*P*_ and *R*_*H*_ denotes the real-runtime produced by PIRN and Hybroscale, respectively. Figure 13, showing the distribution of the speedups corresponding to each of those tree sets, reveals that for three input trees Hybroscale is on mean average about 110 times faster than PIRN, for four input trees on mean average about 170 times, and for five input trees on mean average about 190 times.

### Application to a grass dataset

As mentioned above our algorithm computes all representative networks for a set of input trees. In particular, given only two input trees, this means that Hybroscale in general outputs multiple networks for each maximum acyclic agreement forest instead of only one as it is the case for the method described in the work of Albrecht*et al.* [9]. As a consequence, the output usually consists of a huge number of different hybridization networks, which is demonstrated by Tables 1 and 2 presenting the results of our software Hybroscale applied to a well known grass (*Poaceae*) dataset^3^ consisting of three nuclear loci and three chloroplast genes. This dataset, which is also used in the work of van Iersel *et al.* [20], was originally published by the Grass Phylogeny Working Group (2001) and reanalyzed in Schmidt (2003).

Again, we ran Hybroscale on a grid computer providing 16 cores and 40 GB RAM for each tree set within the grass dataset and summarized the respective results in Table 2. This table shows that Hybroscale is able to calculate the hybridization number for 50 out of 57 tree sets. This means, in particular, that for seven tree sets Hybroscale cannot produce a result within a time limit of 20 minutes. Moreover, even though for 5 tree sets the hybridization number could be calculated, the respective entire set of representative networks could not be calculated as in this case a time limit of 20 minute is not sufficient to explore the whole solution space. Consequently, this biological example demonstrates that, although our algorithm seems to be faster than all so far existing methods, calculating minimum hybridization networks remains a computationally hard problem, which is still not solved sufficiently.

In Fig. 7, one out of 324 possible hybridization networks reconciling four different binary phylogenetic trees corresponding to the sequences *ndhf*, *rpoC*, *waxy*, and *ITS* is given. The embedding of the trees is demonstrated by the four colors blue, red, green, and orange. This means, for example, that we can simply determine the embedding of the tree corresponding to *rpoC*, which is denoted as *Tree 1* in this case, by taking the red colored edges into account. Moreover, the support values assigned to each hybridization node reveal that a hybridization event involving the two species *oryza* and *lygeum* occurs in 97 *%* of all 324 networks, which could be a strong signal that this event is also part of the true underlying evolutionary history. However, the reader should be aware of the fact that there still exist other mechanisms explaining such inconsistencies, as for example incomplete lineage sorting. Hence, such networks just help to build hypothesis that still have to be tested by applying further experiments.

## Conclusion

As already discussed in the work of Albrecht *et al.* [9], it makes sense to consider hybridization if there is a significant difference between certain gene trees and if other effects, as for example incomplete lineage sorting, could be excluded. The number of genes affected by hybridization, however, is of course not limited to a fixed value, e.g., two, and, thus, a method computing hybridization networks for an arbitrary number of input trees is of high interest.

While some approaches only focus on reconciling two binary phylogenetic $\mathcal {X}$-trees [9, 21], in this article, we present the algorithm ALLHNETWORKS that is able to cope with multiple input trees. Moreover, instead of reporting just the hybridization number or only a small number of hybridization networks, our approach is based on the first algorithm that is able to output all representative networks, which is an important feature enabling the computation of meaningful support values indicating which of the computed hybridization events might have played an important role during evolution. Additionally, in combination with our software Hybroscale, we improve the interpretation of the reported hybridization networks by assigning support values to each hybridization node and by highlighting the embedding of all input trees.

Additionally, our reported simulation study indicates that our algorithm is much faster than the only so far existing software PIRNv2.0 [7, 8] for computing the exact hybridization number for more than two binary phylogenetic trees on the same set of taxa. As shown in Fig. 12, the better performance is not only due to parallelization but apparently also due to algorithmic issues and, presumably, due to the application of certain reduction rules.

Finally, we would like to mention that in the meantime we have extended the algorithm ALLHNETWORKS such that it can be applied to multiple rooted *multifurcating* phylogenetic trees sharing an *overlapping* set of taxa. Moreover, to make Hybroscale applicable to larger input sizes, we have added an option allowing to generate certain constraints for either limiting the search space of all representative networks before running our algorithm or to filter the set of reported networks after its computation. This mechanism was motivated by the previous work of Kelk *et. al* [22] suggesting to come up with a general method for generating these constraints, which is absolutely meaningful in our point of view.

## Availability and requirements

- **Project name:** Hybroscale
- **Project home page:** www.bio.ifi.lmu.de/softwareservices/hybroscale
- **Operating system(s):** Platform independent
- **Programming language:** Java
- **Other requirements:** Java 7 or higher
- **Any restrictions to use by non-academics:** none

## Endnotes

^1^ www.bio.ifi.lmu.de/softwareservices/hybroscale

^2^ www.bio.ifi.lmu.de/softwareservices/hybroscale

^3^ www.sites.google.com/site/cassalgorithm/data-sets

## Additional file

Additional file 1 **Supplementary material.** Supplementary Material contains Supplementary Figures.

## References

[1] Mallet J (2007). Hybrid speciation. Nature.

[2] Rieseberg LH (2000). Hybridization, introgression, and linkage evolution. Plant Mol Biol..

[3] Soltis P, Soltis D (2009). The role of hybridization in plant speciation. J Comput Biol..

[4] Schwenk K, Brede N, Streitl B (2008). Introduction. extent, processes and evolutionary impact of interspecific hybridization in animals. Phil Trans R Soc B Biol Sci..

[5] Bordewich M, Semple C (2007). Computing the minimum number of hybridization events for a consistent evolutionary history. Discrete Appl Math..

[6] van Iersel L, Linz S (2013). A quadratic kernel for computing the hybridization number of multiple trees. Inform Process Lett..

[7] Wu Y (2009). Close lower and upper bounds for the minimum reticulate network of multiple phylogenetic trees. Bioinformatics.

[8] Wu Y (2013). An algorithm for constructing parsimonious hybridization networks with multiple phylogenetic trees. J Comput Biol..

[9] Albrecht B, Scornavacca C, Cenci C, Huson DH (2011). Fast computation of minimum hybridization networks. Bioinformatics.

[10] Baroni M, Semple C, Steel M (2006). Hybrids in real-runtime. Syst Biol..

[11] Bordewich M, Semple C (2007). Computing the hybridization number of two phylogenetic tress is fixed-parameter traceable. IEEE/ACM Trans Comput Biol Bioinformatics.

[12] Whidden C, Beiko R, Zeh N, Festa P (2010). Fast FPT algorithms for computing rooted agreement forests: Theory and experiments. In: Proceedings of the 9th International Symposium on Experimental Algorithms, SEA 2010.

[13] Albrecht B. Computing hybridization networks for multiple rooted binary phylogenetic trees by maximum acyclic agreement forests. arXiv:1408.3044. 2014.

[14] Huson DH, Rupp R, Scornavacca C (2011). Phylogenetic Networks: Concepts, Algorithm and Applications.

[15] Baroni M, Gruenewald S, Moulton V, Semple C (2005). Bounding the number of hybridisation events for a consisten evolutionary history. Math Biol..

[16] Bordewich M, Semple C (2005). On the computational complexity of the rooted subtree prune and regraft distance. Ann Combinator..

[17] Linz S (2008). Reticulation in evolution.

[18] Scornavacca C, Linz S, Albrecht B (2010). A first step towards computing all hybridization networks for two rooted binary phylogenetic trees. J Comput Biol..

[19] Cardona G, Rossello F, Valiente G (2008). Extended newick: It is time for a standard representation of phylogenetic networks. BMC Bioinformatics.

[20] Kelk S, Rupp R, Huson DH, van Iersel L (2010). Phylogenetic networks do not need to be complex: using fewer reticulations to represent conflicting clusters. Bioinformatics.

[21] Chen ZZ, Wang L (2010). Hybridnet: a tool for constructing hybridization networks. Bioinformatics.

[22] Kelk S, Linz S, Morrison DA. Fighting network space: it is time for an sql-type language to filter phylogenetic networks. arXiv:1310.6844. 2013.

