# Supplementary material for: Computing all hybridization networks for multiple binary phylogenetic input trees
Source: BMC Bioinformatics. 2015 Jul 30;16:236. doi: 10.1186/s12859-015-0660-7 (PMC4518679; doi:10.1186/s12859-015-0660-7)
Supplement: Supplementary file 1 — Supplementary material. Supplementary Material contains Supplementary Figures. [file 12859_2015_660_MOESM1_ESM.pdf]

SUPPLEMENTARY MATERIAL

Computing all hybridization networks for multiple binary phylogenetic input trees

Supplementary Figures

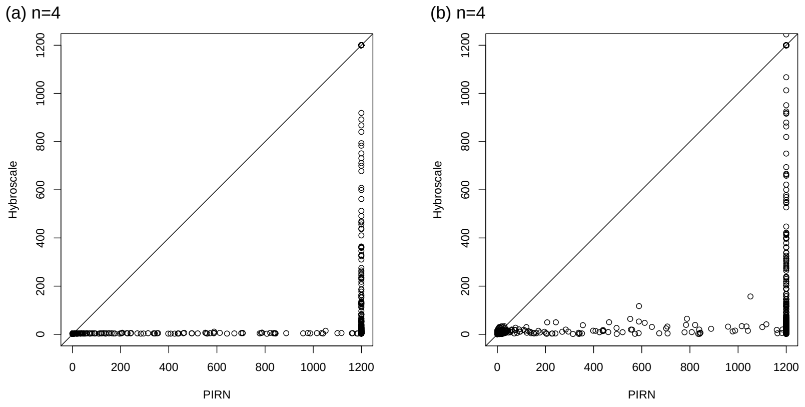

Supplementary Figure 1 A scatterplot of the runtimes generated by PIRN (x-axis) against the runtimes generated by Hybroscale (y-axis) of all 810 data sets consisting of *four* input trees. The plot is generated for the real-time **(a)** and the user-time **(b)** of Hybroscale. Note that PIRN is not able to compute the result for 578 tree sets corresponding to each dot in the figure whose x-value is 1200. From those tree sets just 103 could not be computed by Hybroscale.

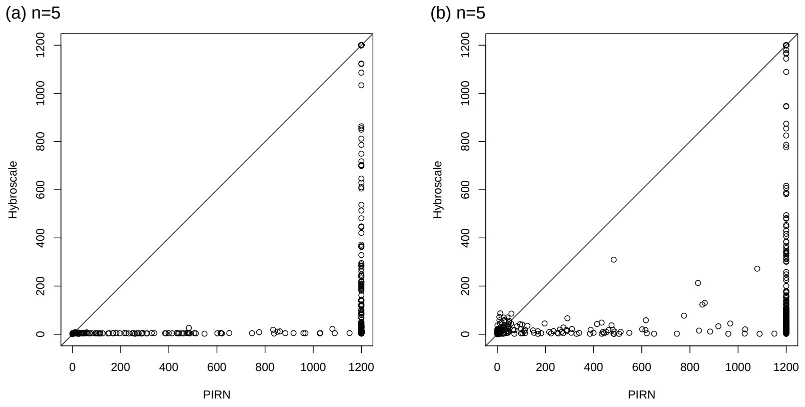

Supplementary Figure 2 A scatterplot of the runtimes generated by PIRN (x-axis) against the runtimes generated by Hybroscale (y-axis) of all 810 data sets consisting of *five* input trees. The plot is generated for the real-time **(a)** and the user-time **(b)** of Hybroscale. Note that PIRN is not able to compute the result for 623 tree sets corresponding to each dot in the figure whose x-value is 1200. From those tree sets just 224 could not be computed by Hybroscale.

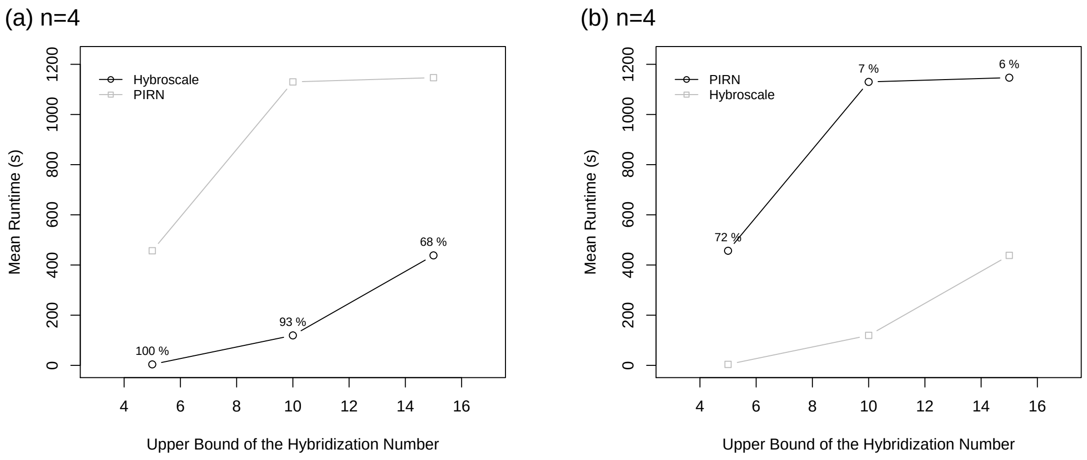

Supplementary Figure 3 Figure **(a)** on the left hand side corresponds to Hybroscale and figure **(b)** on the right hand side to PIRN. The two figures show the average runtime grouped by parameter k, the upper bound of the hybridization number. Each percentage indicates the proportion of tree sets which could be computed within the time limit of 20 minutes.

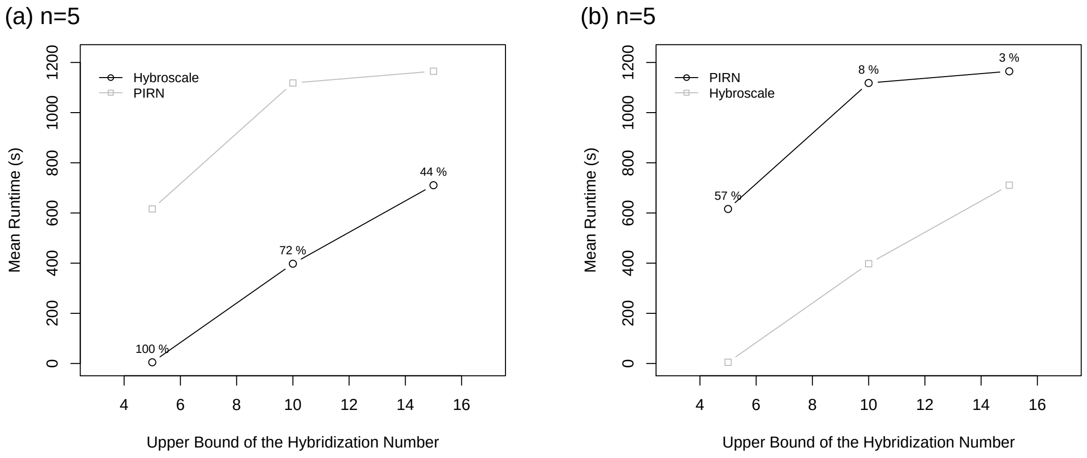

Supplementary Figure 4 Figure **(a)** on the left hand side corresponds to Hybroscale and figure **(b)** on the right hand side to PIRN. The two figures show the average runtime grouped by parameter k, the upper bound of the hybridization number. Each percentage indicates the proportion of tree sets which could be computed within the time limit of 20 minutes.

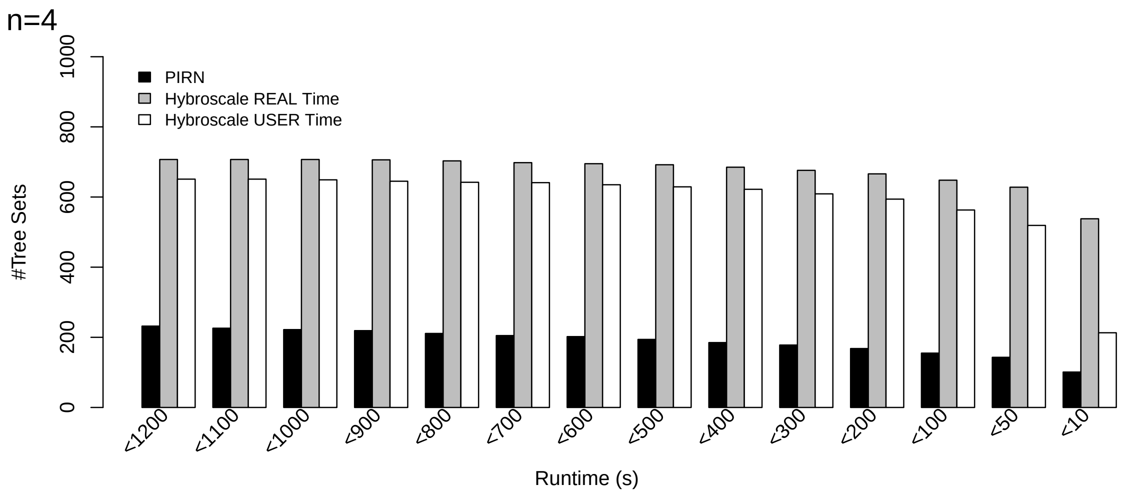

Supplementary Figure 5 The figure shows the number of tree sets that could be computed within the runtime given at the x-axis by considering the real-runtime of PIRN and Hybroscale and, additionally, the user-runtime of Hybroscale.

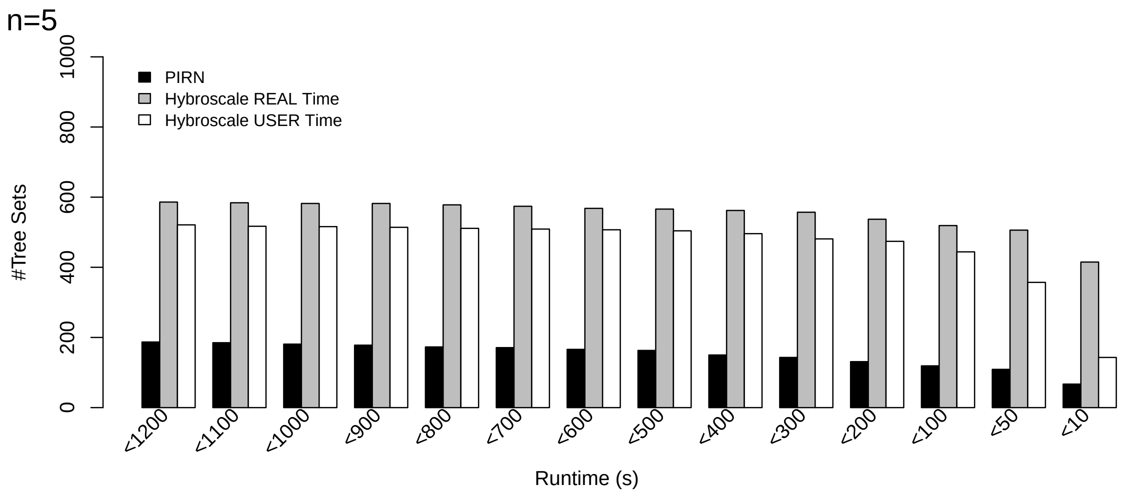

Supplementary Figure 6 The figure shows the number of tree sets that could be computed within the runtime given at the x-axis by considering the real-runtime of PIRN and Hybroscale and, additionally, the user-runtime of Hybroscale.

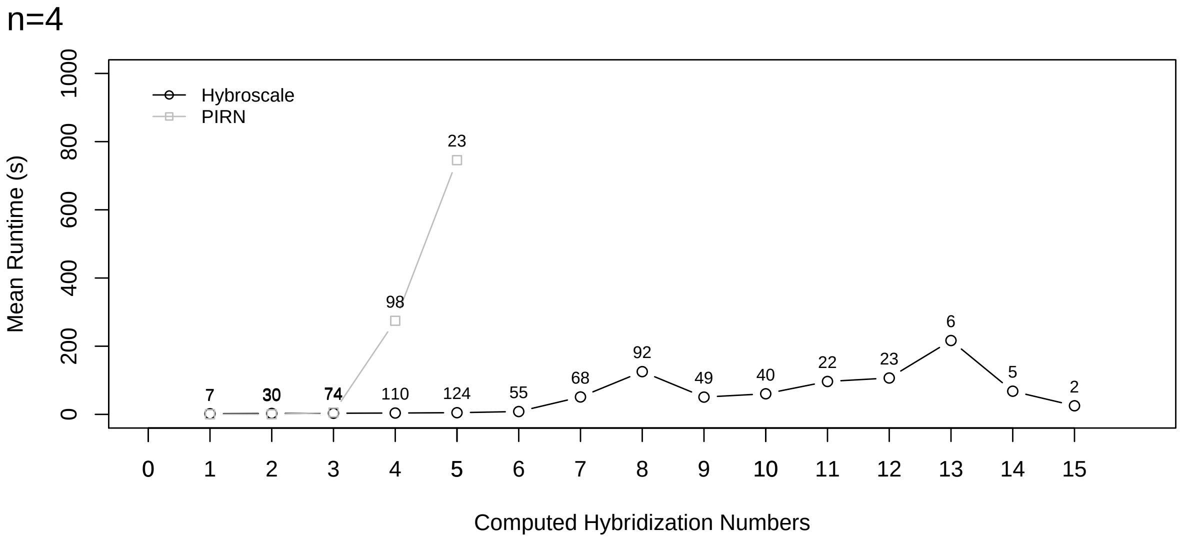

Supplementary Figure 7 The figure shows the average runtime of all tree sets grouped by the computed hybridization numbers. The numbers inside the plot indicate how many tree sets could be computed for the corresponding hybridization number within the time limit. Note that for the hybridization numbers 0 to 3 this could be achieved by both programs for all corresponding tree sets.

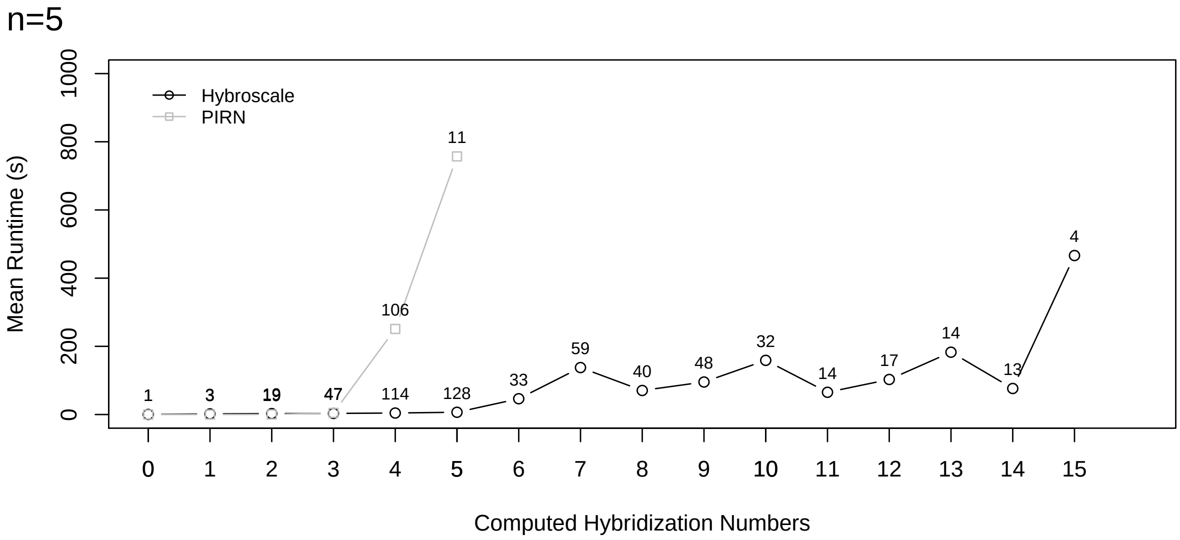

Supplementary Figure 8 The figure shows the average runtime of all tree sets grouped by the computed hybridization numbers. The numbers inside the plot indicate how many tree sets could be computed for the corresponding hybridization number within the time limit. Note that for the hybridization numbers 0 to 3 this could be achieved by both programs for all corresponding tree sets.
